# What Works for Promoting Health at School: Improving Programs against the Substance Abuse

**DOI:** 10.3389/fpsyg.2017.01743

**Published:** 2017-10-12

**Authors:** Elena Faccio, Antonio Iudici, Francesca Turco, Matteo Mazzucato, Gianluca Castelnuovo

**Affiliations:** ^1^Department of Philosophy, Sociology, Education and Applied Psychology, University of Padua, Padua, Italy; ^2^Istituto Auxologico Italiano IRCCS, Psychology Research Laboratory, Ospedale San Giuseppe, Verbania, Italy; ^3^Department of Psychology, Catholic University of the Sacred Heart, Milan, Italy

**Keywords:** health programs at school, methodology, substance abuse, Symbolic Interactionism

The school is one of the most important contexts for carrying out health promotion programs related to the abuse of substances. Over the years, methods and intervention models have changed a great deal, both in relation to the evolution of health goals and to the role played by experts, students, parents, and teachers. We would like to offer a different perspective on health promotion at school by discussing the weaknesses and strengths of the most used methods, in order to identify the appropriate methodology, based on recent evidence research findings. We used Scopus as database for reviewing existing literature. The evolution in the methodology of health promotion programs can be synthesized through a sequence of three phases, from the 1960s to the present day.

## First phase: health education centered on the figure of the expert (early 1960s to early 1970s)

This method of prevention has been in vogue for many years and is still popular. It was based on the idea that an individual with medical and scientific expertise was the ideal partner for any prevention intervention. It envisages delegating to the expert the tasks of designing, implementing, conducting, and evaluating the program. It emphasized the medical and scientific knowledge of the individual who came from the Institute of Mental Health and Addiction into the school and was viewed as an expert in substance abuse, but who knew very little about the students.Since s/he was not an educator, often the external expert found difficulties when it came to mediating his/her technical language with the needs of the students he was talking to. These interventions were, therefore, mainly mini-conferences for giving information (often presented as “shock information”, Toumbourou, 2007; Cimini et al., 2009), and were not adapted to the specific needs of the groups to which they were addressed.

The interventions so developed are focused on “problematic” symptoms or behaviors, considered as “causes” and predictors of drug consumption that is conceived of as an internal and almost innate tendency. This methodological choice depends on the theoretical framework which considers “addiction” as coming from people of interior fragility and which is reducible by increasing awareness of the dangers and risks (Evans-Whipp et al., 2007). In contrast to this trend, the literature has been trying to prove how this choice of approach might be ineffective (Bangert-Drowns, 1988) for many reasons: it fully leaves out the personal student standpoints, beliefs, and meanings about these kinds of experience (Vander Leanen, 2011); students are passive and they do not participate in the construction of the program (Orsini et al., 2012), teachers are little involved in the intervention, and remain an unexploited resource (Van Hout, 2012), and their knowledge about the classes rarely becomes a starting point for calibrating the activities (Pettigrew et al., 2012).

## Second phase: health education centered on the figure of the teacher with ad hoc training (1970s to 1980s)

Many schools soon realized that a work highly focused on the expertise of the external expert, based on “spot” intervention, was not able to touch the real student needs, as the continuity of the relationship is the main educational tool. The effectiveness of preventive intervention cannot ignore the daily life of the relationship and the relationship between educator and student. Consequently, a strong movement was born that was devoted to the culture of the direct promotion of health within the system itself. This has led to the development of a “health promoting school” through new teaching methodologies and innovative learning processes, including so-called *life skills* and *cooperative learning*. These methodologies can only be brought by teachers who have received ad hoc training, and are strongly motivated to use their teaching role to coordinate an effective strategy that allows for the redistribution of tasks and functions: students are educating students and the role of the expert is often replaced by testimony, or by someone who, having a history of substance consumption, can conjure up emotions, offer advice and dissuade students from risk behaviors and dangerous situations (Pellai, 2006; Iudici, 2015; Cipolletta et al., 2017).

## Third phase: health education focused on students' skills, or models related to the practice of peer education (from the 1990s to the present day)

Peer education is a methodology that, at different levels, involves teenagers as the main actors in the strategic and operational choices with regard to prevention projects (Vygotskij, 1980; Gardner, 1993). There are multiple applications of this methodology: from the pure model, where peer educators and theme are chosen by adults, to the mixed model where the theme of work is chosen by adults while its realization is in the hands of adolescents; to the empowered peer education, where adolescents exercise decision-making power in the same way as adults.

This methodology focuses on the social skills of the young educators. It eliminates the traditional contribution of the “teacher,” leaving almost a blank paper to the peers. In doing so, the effectiveness of the project is closely linked to the charisma and popularity of the peer educators. The contribution of the peer educators is also more enigmatic, based on their relationship to substances and their particular point of view.

## How should the dissemination of effective programmes be conducted?

Cuijpers (2002) conducted a systematic literature review examining the current scientific knowledge with regard to the characteristics of effective drug prevention programs. The most important evidence-based quality criteria identified in these researches relate to interactive delivery methods, the role of peer leaders, the development of life skills, and the emphasis on social norms, commitment not to use, and intentions not to use. The increase in effectiveness is therefore linked to the ability of the program to enter into the adolescents' way of thinking, into their beliefs, and to strengthen resistance to social pressures. More extensive community intervention and also the involvement of teachers, parents, and the entire community has been proven to be the best method to strengthen the effects.

## A new methodological proposal for intervention

Since the methodology chosen for conducting an intervention is linked to precise theoretical assumptions and is strictly bound to the aims of the intervention program, it is important to ascertain the strength of each of the considered methodologies, on the basis of a scientific approach. Referring to the theoretical model of Symbolic Interactionism (Mead, 1934; Blumer, 1962) the process of producing meanings does not dwell within the heads of individuals; rather, it is the effect of the discourseful negotiation of meanings and the exchange of points of view (Faccio, 2011; Faccio et al., 2014, 2016, 2017). From this first theoretical assumption, some important implications arise for the work at school:

1. The effectivness of the intervention does not depend on the discussion of specific contents about the substance use, rather, it depends on sharing points of view about what students consider to be meaningful as a form of addiction.
2. Central to project planning should be the person (the student and his network of relationships) in terms of the globality of his expressive and communicative potential, regardless of the presence or absence of symptoms, pathology, or risk factors (Iudici and Verdecchia, 2015).
3. In general terms, we might hypothesize that the proposal that expert should negotiate with the school leader and the professors responsible for health projects—in line with our theoretical model—should involve a shift in conceptualizing the theme of addiction, from the Change in Behavior to the Development of Skills, and from Identification of the Causes of Consumption, to the research of Intentions (Iudici, 2015). The goal is therefore not to demonize the substance use or to scare the students, rather than to think together with them about the search for identity and personal fulfillment needs that can be entrusted improperly by using substances.

The structure of each meeting must be characterized by an extremely flexible methodology, since the effectiveness of the program depends on how the person is living and what s/he is thinking, and not on the aims defined at the table prior to the meeting (Blumer, 1962; Iudici et al., 2015a,b). Flexibility should not come from randomness or the absence of defined goals, but rather from an initial project hypothesis, built on the basis of a careful analysis of demand and of the real group needs. Functional goals have to be redefined therefore, to progressively activated processes. As an example, we might plan a differentiation in the length of the program based on the needs of the classes: for classes that during the first meeting seem to be little involved with respect to the theme, there could be few meetings (3 or 4) aimed at discussing prejudices and stereotypes about reasons for substance use, while for classes most involved, the meetings could be more numerous and specific (up to 8–10).

The interactivity of the program (one of the golden points to guarantee effectiveness) can derive not only from peer education, but also from the attitude used by the adults during the meetings. The expertise of the health professional who interacts with the class cannot be exhausted in terms of his/her knowledge of the use of substances, but should refer to the expert's psychological competence in reading the class-group dynamic and in involving the adolescents in an active role (Faccio et al., 2013; Iudici, 2015). Peers may not be competent in deciding the objects of the program on the basis of the specificity of the class-group (which is another gold-standard, following Pellai, 2006). In our view, it would be important the reintroduction of the experts, considering as experts not only psychologists and toxicologists, but also former substance users (perhaps in the co-presence with a professional), who have followed a specific training to acquire competence in managing the group's dynamics.

In addition, the collaboration and participation of teachers is really important, since their role is often underestimated (Iudici, 2015). Their way of intervention may vary depending on the project activation phase: before the start, they can address the theme of substance use in their subject matter, they can also meet the experts during an early exchange for the co-construction of the project, since they know the class better than anyone else. Finally, after the project, they are in a position to show students the implications of what was discussed with the experts, recalling content, and ideas (Tupper, 2008).

By summing up, all actors of the school system could be involved, according to their role; also parents might participate in a beginning meeting, for presenting them the program and for listening for their requests about the parental role in dealing with the theme of addiction, and than in a final meeting, for giving them a feedback about the project, perhaps managed by the students themselves co-ordinated by the expert. The effectiveness assessment process must necessarily take place within a program of qualitative methodology (interviews or focus groups) and it must be differentiated on the basis of the role of the participant, of the class-driven path, and the specific goals negotiated with the referents and the students themselves but which should cover the objectives set out above in points 1, 2, and 3.

## Conclusions

Through this new contribution it becomes possible to change the traditional way to construct an intervention. The class and the students become the main focus of the activities, with their personal abilities being promoted as part of a collaborative perspective. In order to impact on their way of thinking and “to speak their language” it is necessary that the project be co-constructed by students and their caregivers, in a negotiating relationship with the teachers and operators.

The addictional presence of ex-consumers of substances with specific relational formation, could promote a progressive change in their belief system about this phenomenon, overcoming prejudice and stereotypes about drug users.

## Author contributions

All five authors (EF, AI, FT, MM, and GC) contributed to the drafting phases of the article listed below: 1. Critical Review of Literature, 2. Discussion of proposed ideas, 3. Schedule the Rationale for Opinion, 4. Re-reading the text and editing, 5. Final approval to the version to be published.

### Conflict of interest statement

The authors declare that the research was conducted in the absence of any commercial or financial relationships that could be construed as a potential conflict of interest. The handling Editor declared a shared affiliation, though no other collaboration, with one of the authors, GC.
